# Natural Aldehyde-Chitosan Schiff Base: Fabrication, pH-Responsive Properties, and Vegetable Preservation

**DOI:** 10.3390/foods12152921

**Published:** 2023-07-31

**Authors:** Jiawei Lin, Hecheng Meng, Xiaobing Guo, Zhongsheng Tang, Shujuan Yu

**Affiliations:** 1 School of Food Sciences and Engineering, South China University of Technology, Guangzhou 510640, China; jwlin.sci@hotmail.com (J.L.);; 2 School of Food Science and Technology, Shihezi University, Shihezi 832003, China; 3 College of Food Science and Engineering, Guangdong Ocean University, Yangjiang 529500, China; 4 Chaozhou Branch of Chemistry and Chemical Engineering Guangdong Laboratory, Chaozhou 521011, China

**Keywords:** imine bonds, broccoli, essential oils, antibacterial activity, shelf life

## Abstract

The aim of the present work was to fabricate Schiff base compounds between chitosan and aldehydes and use the resultant aldehyde-chitosan Schiff bases for broccoli preservation. Using an element analyzer, the degree of substitution was calculated as 68.27–94.65%. The aldehyde-chitosan Schiff bases showed acidic sensitivity to rapid hydrolysis for releasing aldehyde at a buffer solution of pH 4–6, in which more than 39% of the aldehydes were released within 10 h. The release of aldehydes endows the aldehyde-chitosan Schiff bases with a better antibacterial activity at pH 5 than at pH 7. In a simulated CO_2_ (5–15%) atmosphere with high humidity (92%), the hydrolysis of imine bonds (C=N) was triggered and continuously released aldehyde, even without direct contact with the aqueous phase. The application of aldehyde-chitosan Schiff bases significantly extended the shelf life of broccoli from 4 d to 5–7 d and decreased the weight loss of broccoli during storage. In summary, the fabrication of aldehyde-chitosan Schiff bases and the strategy of using pH-response imine bond (C=N) hydrolysis (thus releasing aldehyde to kill microorganisms) were feasible for use in developing EO-incorporated intelligent food packages for vegetable preservation.

## 1. Introduction

The wasting rate of fruit and vegetables in some countries and regions has been estimated at up to 45% [1], despite the fact that there are approximately 828 million people globally who suffer from starvation [2]. Modified atmosphere packaging [3], low-temperature storage [4], membrane (film) coating preservation [5], and chemical preservatives [6] are widely used techniques for fruit and vegetable preservation. As a kind of physical preservation technique, low-temperature preservation is easy to operate and cost-efficient, and is the most commonly used technique in home and industry, but it is unable to deactivate microorganisms. Chemical preservatives (sodium benzoate, NaNO_2_, SO_2_, etc.) can significantly inhibit the growth of microorganisms, but safety concerns still hinder their application. A growing interest in natural preservatives from plants or animals that can be used against food spoilage and extend the shelf life of food has developed because they are safe, eco-friendly, and biodegradable agents [7].

Essential oils (EOs) are volatile, natural, complex compounds characterized by a strong odor that are formed by aromatic plants as secondary metabolites [8]. They are usually obtained by steam- or hydro-distillation first developed in the Middle Ages by Arabs. It is proven that EOs have excellent antioxidant and broad-spectrum antibacterial properties, and they are widely reported as a natural preservative for various food products such as meat, vegetables, fruits, and dairy products [9]. EOs and compounds derived from EOs have been classified by the United States Food and Drug Administration (FDA) and the European Commission (EC) as Generally Recognized as Safe (GRAS) [10]. By penetrating the exopolysaccharides and the phospholipid bilayer, EOs disrupt the conformation and increase the permeability of bacterial cell membranes, thus inducing the leakage of intracellular material and inhibiting the growth of bacterial strains [11]. Karoui and Hassoun [12] found that basil EO showed strong antimicrobial activity to delay spoilage [9] of Atlantic mackerel (*Scomber scombrus*), while rosemary EO showed antioxidant activity to prevent oil oxidation. In addition to the intense aroma, EOs have poor water solubility, high volatility, and are susceptive to light, heat, and oxygen, which has limited their application in the food industry [9]. To surmount the above limitations, several strategies have been developed to improve the stability and the efficiency of EOs, including emulsion encapsulation [13], microencapsulation [14], nanoencapsulation [15,16,17], and film incorporation [18], which could significantly extend the shelf life of food products and maintain food quality. However, all these strategies are dedicated to slowing down the release of EOs, but they are unable to control the release of the active substances according to the actual demand. No matter the food quality changes, the release of EOs will occur, and the release of the active substances is decay exponential. The premature consumption of EOs not only weakens their antibacterial properties, but also wastes a large amount of EOs, hindering their real applications. The development of a cost-efficient technique for stabilizing and modulating EO release is urgently needed to exert their antibacterial properties on demand.

Respiration is the main metabolic activity of vegetables after harvest; it continuously consumes O_2_ and nutrients, while excreting water and CO_2_. When vegetables are sealed in a package, the accumulated CO_2_ reacts with water vapor to form an acidic microenvironment in the package headspace [19]. In recent years, intelligent food packages with pH-response color change properties were designed for freshness monitoring of foods such as seafood [20], poultry [21], and vegetables [19]. For example, methylcellulose film with bromothymol blue methyl red was used as an indicator label to monitor pepper decay [19]. Due to the increased carbon dioxide concentrations in the package as a result of the deterioration of the pepper, the label accurately responded to the pepper freshness with a significant color change from yellow-green to orange. In another work, sugarcane skin anthocyanin was incorporated into cellulose film, which showed bioamine-response properties as color changes from dark green to brown-yellow [20]. The corresponding color change in the food packaging labels enables humans to quickly judge freshness with the naked eye. However, these studies focused on the monitoring of food freshness with a pH-response strategy but not on preservation to extend the shelf life.

The imine bond (C=N), known as the Schiff base structure, is formed by condensation of a carbonyl compound and an amino compound, and shows good toleration at neutral and alkaline conditions but is easily hydrolyzed in acidic media [22]. Previous studies on the food application of chitosan-based Schiff base compounds mainly focused on the enhanced antibacterial activity of newly formed imine bonds. For example, Heras-Mozos et al. [23] proposed that a film composed of chitosan-hydroxybenzaldehyde Schiff bases could significantly inhibit the growth of microorganisms in fruit. But the acid-response hydrolysis properties have not been used to trigger the release of antibacterial substances.

Based on the acidic sensitivity of Schiff bases and the acidic microenvironment in the vegetable package headspace, chitosan (rich in amino groups) was selected as a carrier to synthesize aldehyde-chitosan Schiff bases by reacting with five naturally occurring aldehydes (cinnamaldehyde, CA; benzaldehyde, BZ; vanillin, VL; citral, CT; citronellal, CN) from EOs. It is a novel way to stabilize the volatile aldehydes that could also realize the controlled release of antibacterial aldehydes as triggered by the exposure to CO_2_ and H_2_O from vegetable respiration. First, the reactivity and structural properties of chitosan with different aldehydes were characterized. Then, the pH-response hydrolyzation properties of the aldehyde-chitosan Schiff bases and their antibacterial activities were studied. Finally, the fabricated aldehyde-chitosan Schiff bases were used for broccoli preservation. This work provides for the feasibility of developing an EO-incorporated intelligent food package with pH-response release properties.

## 2. Materials and Methods

### 2.1. Materials

Chitosan (CS, deacetylation degree ≥ 99%) and KBr were purchased from Sigma-Aldrich Co. Ltd. (Shanghai, China). Cinnamaldehyde (CA, purity ≥ 99.9%), benzaldehyde (BZ, purity ≥ 99.9%), vanillin (VL, purity ≥ 99.9%), citral (CT, purity ≥ 99.9%), and citronellal (CN, purity ≥ 99.9%) were the product of Macklin Inc. (Shanghai, China). Staphylococcus aureus (*S. aureus*) (ATCC65389) was the product of Guangdong Institute of Microbiology (Guangzhou, China). The LB Broth was purchased from Guangdong Huankai Microbial SCI&TECH Co. Ltd. (Guangzhou, China). Other unmentioned chemicals were analytically pure.

### 2.2. Synthesis of the Aldehyde-Chitosan Schiff Bases

The synthesis of the aldehyde-chitosan Schiff bases was performed according to the previous report [24] using a direct mixing method. Briefly, 5 g of CS powder was dispersed into 100 mL methanol and magnetically stirred (250 rpm, C-MAG stirrer, IKA Corp., Staufen, Germany) at 25 °C for 18 h. Then, 20 g of aldehyde compounds was added to the CS dispersion. The mixture was sealed, heated with a water bath (55 °C), and magnetically stirred for 24 h. After cooling down to room temperature, the solid substances were collected, and the unreacted aldehyde was removed using Soxhlet extraction. Briefly, the aldehyde-chitosan Schiff base powder was wrapped in filter paper and then immersed in ethanol. The round-bottomed flask (with ethanol) was heated to 70 °C. Siphoning was performed at least 10 times to completely remove the unreacted aldehyde. The resultant aldehyde-chitosan Schiff bases were dried in a vacuum oven at 40 °C to remove the ethanol solvent and unreacted aldehyde, and then stored at −20 °C for analysis.

### 2.3. Characterization

#### 2.3.1. Degree of Substitution

The degree of substitution (*DS*) was determined with an element analyzer (Elementar vario MACRO cube, Elementar Corp., Frankfurt, Germany). The content of *C* and *N* was recorded, and the *DS* was calculated with Equation (1) [25]:

(1) $$DS=\frac{\left(C/N\right)-{\left(C/N\right)}_{0}}{n}$$

where (*C*/*N*)_0_ and (*C*/*N*) are the carbon-to-nitrogen ratio of CS and the fabricated aldehyde-chitosan Schiff bases and *n* is the number of carbons in aldehyde, which is 8, 9, 10, 7, and 10 for VL, CA, CN, BZ, and CT, respectively. Also, the degree of deacetylation in the original CS was calculated as 99.1% according to the literature [25].

#### 2.3.2. Fourier Transform Infrared Spectroscopy (FTIR)

The FTIR spectra of the aldehyde-chitosan Schiff bases were determined with a spectrophotometer (Vector 70, Bruker Corp., Billerica, MA, USA). The sample powder and KBr mixture were pressed as tablets and then recorded at room temperature in the range of 400–4000 cm^−1^ [26,27].

### 2.4. pH-Response Hydrolysis of Aldehyde-Chitosan Schiff Bases

#### 2.4.1. Hydrolysis within the Aqueous Phase

Aldehyde-chitosan Schiff bases (0.1 g) were dispersed into 100 mL buffer solution (50 mM) with pH levels of 4.0 (acetate buffer), 5.0 (acetate buffer), 6.0 (phosphate buffer), and 7.0 (phosphate buffer) at room temperature. The dispersion was magnetically stirred (250 rpm) for 30 h. When the imine bond (C=N) in the Schiff bases was broken, the aldehyde was released into the water and determined as described below.

Aldehyde was determined using high-performance liquid chromatography (HPLC) equipped with a 2998 photodiode array detector (Waters Corp., Milford, MA, USA). The dispersion (1.0 mL) was mixed with 10 mL ethyl acetate, then subjected to vortex mixing for 15 s and quiescence for 1 h to complete the phase separation. The aldehyde-ethyl acetate solution (5 μL) was injected into a C18 column (Atlantis^®^ T3, 5 μm, 4.6 mm × 250 mm, Waters Corp., Milford, MA, USA) with acetonitrile: water (80:20, *v*/*v*) as mobile phase at 0.4 mL/min and 30 °C. The wavelengths used for quantification were 291, 248, 310, 237, and 210 nm for cinnamaldehyde, benzaldehyde, vanillin, citral, and citronellal, respectively. The standard curves of different aldehydes were established using purchased aldehyde standard substances with the same HPLC measurement. The obtained calibration curve of each aldehyde was listed as follows: y = 148.3x + 0.012 (R^2^ = 0.9998, CA); y = 468.1x + 0.041 (R^2^ = 0.9999, BZ); y = 238.6x + 0.035 (R^2^ = 0.9999, VL); y = 238.9x + 0.015 (R^2^ = 0.9998, CT); y = 324.3x + 0.009 (R^2^ = 0.9999, CN).

#### 2.4.2. Hydrolysis within the High Humidity and CO_2_ Atmosphere

A high humidity and CO_2_ atmosphere was used as the simulated environment to investigate the hydrolysis of the aldehyde-chitosan Schiff bases. The humidity (93.58%) was controlled with a saturated KNO_3_ solution. The Schiff base (0.2 g) powder was wrapped in filter paper and placed in a sealed plastic box. The saturated KNO_3_ solution was contained in an open beaker and did not contact the Schiff base. The plastic box was sealed with CO_2_ (5%, 10%, and 15%) and stored at 25 °C. The gas with controlled composition was filled using a modified atmosphere packaging machine (LQ-370, Zhejiang Liqiang Packaging Technology Co., Ltd., Wenzhou, China). The concentration of CO_2_ was monitored using the probe equipped with the packaging machines. Due to the fact that the release of aldehyde is very slow, the hydrolysis of the aldehyde-chitosan Schiff bases was analyzed every 12 h using the element analyzer as described in Section 2.3.1.

### 2.5. Antibacterial Activity

*Staphylococcus aureus* was cultivated on LB agar plates at 37 °C for 24 h; then, a single colony was selected to incubate with LB broth (37 °C) until the logarithmic stage. The bacterium suspension was diluted according to Mew’s turbidimetry to 0.5 units (1.5 × 10^8^ CFU/mL). The diluted bacterium suspension (200 μL) was mixed with 20 mL LB broth at varying pH (5.0 and 7.0 with phosphate buffer) and 100 mg aldehyde-chitosan Schiff bases, and then the mixture was incubated at 37 °C with shaking. The OD_600nm_ of the bacterium suspension was determined with a microplate reader (Labserv K3, Thermo Fisher Instrument Co., Ltd., Waltham, MA, USA).

### 2.6. Broccoli Preservation Test

The fresh-harvest broccoli (300 g) was sealed with the aldehyde-chitosan Schiff bases (0.3 g packed in filter paper to avoid direct contact) in a plastic case (1.2 L). The broccoli was stored at 25 °C for 9 d. During the storage, the appearance of the broccoli was observed and the weight loss was determined every day. To evaluate the growth of microorganisms on the surface of the broccoli, 50 g of broccoli (stored for 5 d) was washed with 100 mL sterile water. The water was treated by decimal dilutions for the spread plate. 100 μL diluted water was spread onto the dextrose peptone agar plate and cultivated at 37 °C for 24 h for observation.

### 2.7. Statistical Analysis

Unless otherwise stated, experiments were performed in triplicate, and the data were analyzed using Origin software (OriginPro learning edition) with a one-way analysis of variance (ANOVA) and Scheffe’s test (*p*-value < 0.05).

## 3. Results and Discussion

### 3.1. Reaction between Aldehyde and Chitosan

Due to the complexity of essential oils (EOs), it is hard to analyze the reaction between raw EOs and chitosan (CS). In this work, five naturally occurring aldehyde compounds (cinnamaldehyde, CA; benzaldehyde, BZ; vanillin, VL; citral, CT; citronellal, CN) were selected to fabricate aldehyde-chitosan Schiff bases; these compounds are the main aroma components of cinnamon (*Cinnamomum zeylanicum Blume*) EOs [28], bitter almond (*Prunus amygdalus var. Amara*) EOs [29], vanilla (*Vanilla planifolia Andrew*) EOs [30], *Litsea cubeba* EOs [31], and citronella (*Cymbopogon winterianus Jowitt.*) EOs [32], respectively.

**Figure 1:**
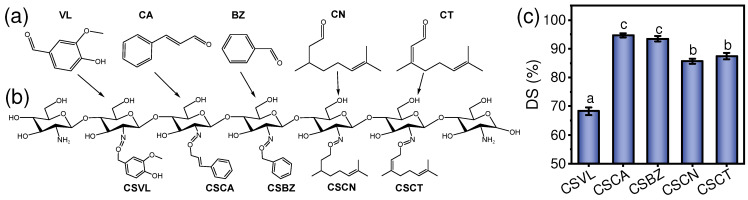
Structure of the aldehyde compounds (**a**) and fabricated aldehyde-chitosan Schiff bases (**b**). The degree of substitution (DS) of varying samples (**c**). In panel (**c**), the different letters on the column (a–c) indicate statistically significant differences (*p* < 0.05) among the samples.

Figure 1a,b depicts the Schiff base reaction mechanism between the free amino group (–NH_2_) of CS and the aldehyde group (–CHO) of the different aldehydes, where the dehydration condensation reaction happens and the imine bond (C=N) is formed. By analyzing the C/N ratio, the degree of deacetylation in CS was calculated as 99.1% (meeting the statement of manufacture), and the degree of substitution (DS) in the aldehyde-chitosan Schiff bases was decreased in order: CSCA (94.65%) > CSBZ (93.48%) > CSCT (87.48%) > CSCN (85.68%) > CSVL (68.27%) (Figure 1c), which is in agreement with the report of Chen et al. [33]. By inducing the Schiff base reaction between aldehydes and CS at the oil–water interface, Chen et al. proposed that the reaction rates were decreased in order: CSCA > CSCT ≈ CSCN > CSVL. They also correlated the reaction rates with the hydrophobicity of different aldehydes. The Schiff base reaction is a dehydration condensation reaction, and the repulsion of the water generated during the reaction can promote the reaction [34]. The higher the hydrophobicity, the stronger the repulsion of the aldehyde and water, thus facilitating the formation of the Schiff base. The solubility of an aldehyde in water is an indicator of its hydrophobicity. By directly dispersing aldehyde compounds into water, the water solubility of the tested samples was decreased in order: VL (15.35 mg/mL) > CA (1.46 mg/mL) > CT (1.38 mg/mL) > CN (0.33 mg/mL) > BZ (0.13 mg/mL). It was suggested that the significantly lower DS of CSVL (68.27%) may be attributed to its extremely higher solubility (15.35 mg/mL). However, CN with lower water solubility than CA and CT showed a lower DS than CA and CT, suggesting that more factors (chemical structure, existence of double bonds and benzene ring) might contribute to the reaction between CS and aldehydes and more in-depth investigations are needed in future work.

**Figure 2:**
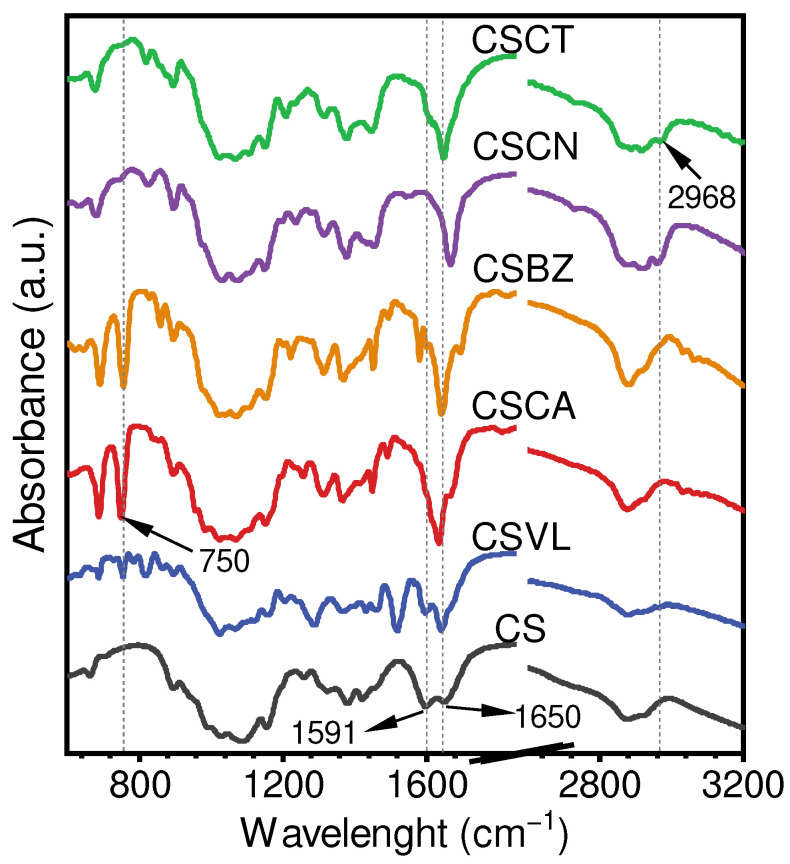
FTIR spectrum of CS and aldehyde-chitosan Schiff bases.

The formation of imine bonds (C=N) was verified with the FTIR spectrum (Figure 2). The absorbance at 1650 cm^−1^ and 1591 cm^−1^ corresponds to the stretching vibrations of the amine I (–C=O) and amine II (–NH) [34]. After the reaction with the aldehyde compounds, all the spectra of the aldehyde-chitosan Schiff bases showed a significant decrease in the amine II, ascribed to the consumption of –NH during the formation of the imine bonds (C=N) [35]. Although decreased significantly, the peak of amine II in CSVL was still obviously stronger than in the other aldehyde-chitosan Schiff bases, which is in agreement with the result of its lower DS (68.27%) (Figure 1c). It was found that the peak of –C=O in all aldehyde-chitosan Schiff bases not only became stronger, but also shifted to the range of 1631~1668 cm^−1^; similar shifting was also observed by Chen et al. [33]. These peaks were assigned to the overlapping of the newly formed C=N and amine I (–C=O), and the shifting was attributed to the conjugation effect [36]. Generally, the conjugation effect induced the red shift of the absorbance peaks. The stronger the conjugation effect, the more obvious the red shift [37]. The peaks of C=N in different aldehyde-chitosan Schiff bases were located at 1631 cm^−1^ (CSCA), 1639 cm^−1^ (CSBZ), 1641 cm^−1^ (CSVL), 1647 cm^−1^ (CSCT), and 1666 cm^−1^ (CSCN). According to the chemical structures, CA, BZ, and VL were aromatic aldehydes, while CT and CN were aliphatic aldehydes (Figure 1a). The imine bonds (C=N) could form a conjugate structure with the adjacent benzene ring and/or double bond [38], and the impact of the benzene ring on the conjugation effect was stronger than the double bond [39]. Therefore, due to the existence of both a benzene ring and a double bond, the red shift of the imine bond peak in CSCA was the most significant. For the spectra of CSVL, CSCA, and CSBZ, the peak at 750 cm^−1^ was assigned to the –CH bending of the aromatic ring [40], indicating the existence of an aromatic ring. Compared with CS, the new absorbance peak at 2968 cm^−1^ in CSCT and CSCN was assigned to the stretching vibration of –CH_2_, which might be induced by the –CH_2_ of the aliphatic hydrocarbon long chain [41]. The above results indicate that aldehyde-chitosan Schiff bases were successfully fabricated.

### 3.2. pH-Response Hydrolysis and Antibacterial Properties of Aldehyde-Chitosan Schiff Bases

**Figure 3:**
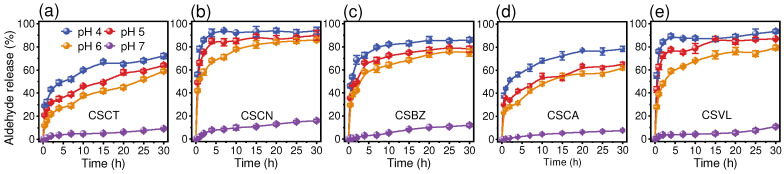
The release of aldehyde from aldehyde-chitosan Schiff bases under varying pH values (pH 4~7). (**a**) CSCT, (**b**) CSCN, (**c**) CSBZ, (**d**) CSCA, and (**e**) CSVL.

The hydrolysis of the aldehyde-chitosan Schiff bases was monitored using the release of aldehyde when exposed to buffer solutions with different pH values (Figure 3). At pH 7, all five aldehyde-chitosan Schiff bases showed a slow increase in aldehyde release within 30 h of the experiment (total release ranging from 8.0% to 16.1%), suggesting relative stability at neutral pH. When the pH decreased to acidic conditions (4–6), the release of aldehyde became significant, more than 30% within 1 h, verifying the pH-response hydrolysis of imine bonds (C=N) to acid. Similar pH-response hydrolysis (hydrolysis at pH 5) properties of imine bonds were also reported for cellulose-derived Schiff base nanoparticles to control the release of drugs [42]. Specifically, CSCN, CSBZ, and CSVL were more sensitive to acid stimulation. Take pH 6 as an instance, the release of aldehyde for CSCN, CSVL, and CSBZ in a 10 h reaction was 78.2%, 68.1%, and 61.3%, respectively, far exceeding that of CSCA (48.3%) and CSCT (39.1%).

**Figure 4:**
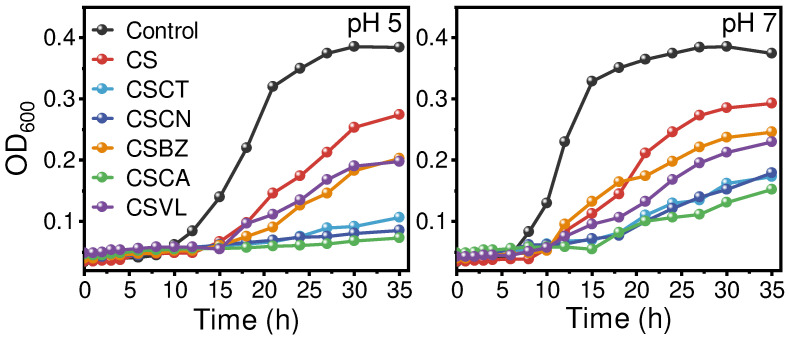
The antibacterial properties of live curves of aldehyde-chitosan Schiff bases on the growth curves of *S. aureus* under pH 5 and pH 7.

In addition to the acid-response hydrolysis properties, aldehyde-chitosan Schiff bases also need to exert a good antibacterial effect to be used for vegetable preservation. In this work, a CO_2_ atmosphere with high humidity formed by vegetable respiration was designed to trigger the hydrolysis of imine bonds (C=N). In our preliminary experiment, when the concentration of CO_2_ in a sealed container exceeded 5%, the pH of the aqueous phase was maintained at around 5. Therefore, the antibacterial properties of the aldehyde-chitosan Schiff bases were tested at pH 5 and pH 7 (Figure 4). Comparing the control at pH 5 and pH 7, acidic conditions inhibited the growth of *S. aureus* because they affected the activity of the microorganisms. CS is a kind of natural polysaccharide with broad-spectrum antibacterial activity [43], but its inhibition of *S. aureus* was inferior to that of the aldehyde-chitosan Schiff bases (no matter what pH), suggesting that the fabrication of Schiff bases by reacting with aldehyde is a feasible way to improve the antibacterial activity of CS (regardless of aldehyde release). Comparing the growth curves of CSVL and CSBZ at two pH values, the values of OD_600_ were similar after 35 h cultivation, implying that their antibacterial properties were not significantly improved when the hydrolysis happened at pH 5. It should be noted that VL and BZ released significantly when exposed to pH 5, which is accompanied by the decrease in imine bonds (C=N). As essential oils, VL and BZ exert antibacterial activity by disturbing the structure of cell membranes [10,11]. The imine bonds (C=N) were stable at pH 7 (Figure 3) and extensively reported with antibacterial properties [44]. Therefore, the similar antibacterial properties of CSVL and CSBZ at pH 5 and pH 7 might be ascribed to the similar antibacterial activity of VL (BZ) and imine bonds (C=N). During the whole cultivation period, the increase in OD_600_ in CSCA, CSCN, and CSCT at pH 5 was slow, and the *S. aureus* dispersion showed a clear and transparent appearance. In comparison, the growth curves at pH 7 were significantly increased and the *S. aureus* dispersion became turbid. Studies have reported that CA, CN, and CT have broad-spectrum antibacterial activity, and their minimum inhibitory concentrations (MICs) to *S. aureus* are 0.31 [45], 0.86 [46], and 0.5 mg/mL [47], respectively. As the main aroma component of cinnamon (*Cinnamomum zeylanicum Blume*) EOs, *Litsea cubeba* EOs, and citronella (*Cymbopogon winterianus Jowitt.*) EOs, respectively, CA, CN, and CT were able to penetrate the exopolysaccharides and phospholipid bilayer and disrupt the conformation, increasing the permeability of the bacterial cell membranes, thus inducing the leakage of intracellular material and inhibiting the growth of the bacterial strains [11].

### 3.3. Hydrolysis of Aldehyde-Chitosan Schiff Bases in CO_2_ with High Humidity

**Figure 5:**
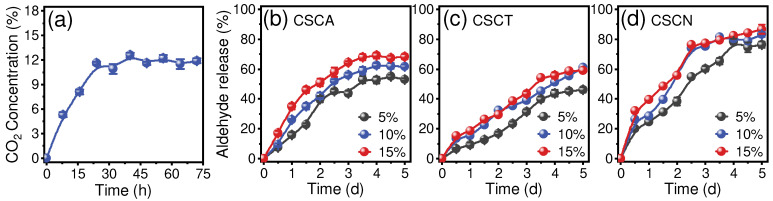
(**a**) Accumulated CO_2_ concentration (%*v*/*v*) of broccoli in a sealed box. The release of aldehyde from CSCA (**b**), CSCT (**c**), and CSCN (**d**) at varying CO_2_ concentrations (5~15%) with high humidity.

To verify that aldehyde-chitosan Schiff bases can be triggered to hydrolyze in the presence of CO_2_ with high humidity, we determined the changes of CO_2_ concentration in a sealed container (1.2 L plastic case) as affected by the respiration of broccoli (200 g). As shown in Figure 5a, CO_2_ concentration rapidly increases to 5.3% and 11.6% at 8 h and 24 h, then reaches a plateau at ~12% as time extends. Accordingly, three CO_2_ concentration gradients (5%, 10%, and 15%) and high humidity (92%, controlled with a saturated KNO_3_ solution) within a sealed container were set as the simulation environment. The responsive hydrolysis of aldehyde-chitosan Schiff bases was tested by avoiding direct contact with water. Because of their superior antibacterial activity (Figure 4), CSCA, CSCN, and CSCT were selected to evaluate their responsive hydrolysis in the simulation environment (Figure 5b–d). The hydrolysis was exactly triggered even though the release of aldehydes was slow because there was no direct contact between the aldehyde-chitosan Schiff bases and the aqueous solution. The release of aldehydes showed a positive correlation with the CO_2_ concentration, and a higher CO_2_ concentration was more conducive to the hydrolysis of imine bonds (C=N). Because the maximum CO_2_ concentration of the sealed broccoli was ~12% (Figure 5a), we took 10% CO_2_ concentration as an instance for analysis. The aldehyde release speed of CSCA and CSCN was faster than CSCT; the release after 3 d storage was 56.6%, 75.2%, and 39.2%, respectively. This trend is similar to the release trend in solution (Figure 3), suggesting that the hydrolysis of aldehyde-chitosan Schiff bases in the simulation environment (within CO_2_ with high humidity) follows similar pH-response hydrolysis characteristics as in aqueous phases.

### 3.4. Broccoli Preservation Study

**Figure 6:**
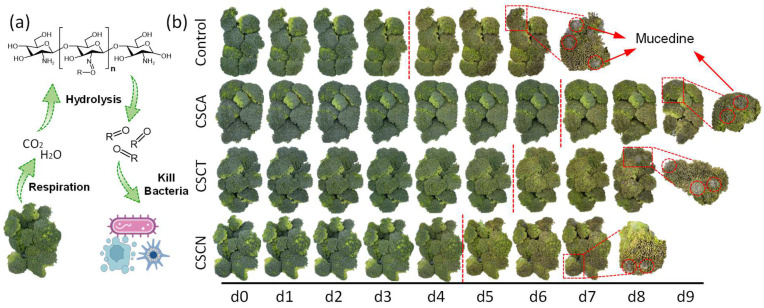
Schematic diagram of aldehyde-chitosan Schiff bases for broccoli preservation (**a**). Visual changes of broccoli during storage as affected by aldehyde-chitosan Schiff bases (**b**).

Broccoli is a nutritious vegetable and is popular in the market. However, broccoli has a high respiration intensity (180–300 mg CO_2_/kg/h, 20 °C) [48] and is intolerant of postharvest storage. After storage at 20 °C for 4 d, broccoli will quickly turn from green to yellow and deteriorate rapidly, accompanied by a rapid decrease in nutritional content [49]. The schematic diagram of aldehyde-chitosan Schiff bases for broccoli preservation is depicted in Figure 6a. When sealed in a package, broccoli with high respiration intensity continuously exhales H_2_O and CO_2_, forming an atmosphere with high CO_2_ and humidity. Although without direct contact with vegetables, the exposure of aldehyde-chitosan Schiff bases to an atmosphere with high CO_2_ and humidity induces the hydrolysis of imine bonds (C=N), thus releasing the aldehyde into the package. The released aldehyde is highly volatile and able to penetrate the surface crevices of the broccoli to kill microorganisms. As shown in Figure 6b, the broccoli in both the control and CS groups turned from green to yellow on day 4, became completely yellow on day 5 (unsuitable for consumption), and perished on day 6 as mucedine appeared on the surface. Although CS has antibacterial activity, without direct contact with the surface of the broccoli, the antibacterial effect cannot be exerted; thus, the shelf life of the broccoli in the CS groups was similar to the control groups. With the use of aldehyde-chitosan Schiff bases, the time for turning color to yellow was delayed from 4 d (control) to 5 d (CSCN), 6 d (CSCT), and 7 d (CSCA), suggesting the shelf life of broccoli extended from 4 d to 5–7 d.

**Figure 7:**
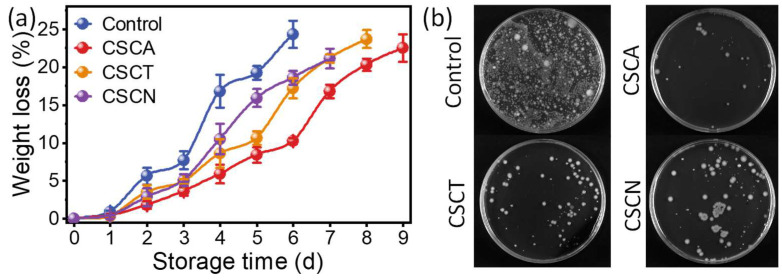
Weight loss of broccoli during storage (**a**). The growth of microorganisms when stored at day 5 (**b**).

Broccoli has a coarse surface and a large specific surface area, endowing it with vigorous transpiration and severe weight loss during storage. As shown in Figure 7, regardless of the shelf life, the weight loss of the different groups was similar (21.1–24.3%) on the day that mucedine appeared (spoilage). At day 4, aldehyde-chitosan Schiff bases significantly decreased the weight loss of broccoli from 16.8% (control) to 10.5% (CSCN), 8.6% (CSCT), and 5.9% (CSCA). It was speculated that the presence of the aldehyde-chitosan Schiff bases played a role in delaying the occurrence of spoilage by inhibiting the growth of microorganisms. As a naturally occurring volatile aromatic substance, essential oils (EOs) are extensively reported as plant-based preservatives [9]. It was believed that the antibacterial activity of aldehyde inhibited the growth of microorganisms, thus extending the shelf life of the broccoli. The growth of microorganisms on the surface of the broccoli was tested with the spread plate count method (Figure 7b). After storage for 5 d, the proliferation of microorganisms in the control was more significant than in the broccoli preserved with CSCA, CSCT, and CSCN. The bacterial colonies are all over the plate in the control sample, but there were only several colonies in the CSCA group. It was speculated that the growth of microorganisms plays an important role in the spoilage of broccoli. The better inhibition of microorganism growth in the CSCA group could be because CA (0.31 mg/mL) has a lower MIC value than CT (0.5 mg/mL) and CN (0.86 mg/mL). Previously, the shelf life of food (both meat and vegetable) could be significantly improved by directly spreading EO emulsions onto the surface of the vegetable [50] or incorporating EOs into a film for food packages [18]. However, direct contact between EOs and food significantly influences the smell and taste of the food. In this work, the strategy of pH-response hydrolysis induced by the CO_2_ and H_2_O from broccoli respiration was designed to trigger the release of aldehyde. Without the high CO_2_ concentration and humidity, the aldehyde-chitosan Schiff bases were stable [51]. When exposed to the CO_2_ and H_2_O from broccoli respiration, imine bonds (C=N) were broken and the aldehyde was released. This strategy not only avoids the direct contact of aldehyde, but also decreases the usage of aldehyde, which would be a feasible strategy to utilize EOs as an effective vegetable preservative.

## 4. Conclusions

Aldehyde-chitosan Schiff bases were fabricated using the reaction between five naturally occurring EOs and chitosan, and were used for broccoli preservation based on the pH-response hydrolysis property. The hydrolysis of aldehyde-chitosan Schiff bases triggered by CO_2_ and H_2_O from broccoli respiration is a novel way to control the release of aldehyde. Aldehyde-chitosan Schiff bases showed the ability to extend the shelf life (from 4 d to 5−7 d) and decrease the weight loss of broccoli. However, how to pack the aldehyde-chitosan Schiff bases and avoid their direct contact with water must be solved in the future to promote their real application. This work provides a novel way for utilizing EOs as vegetable preservatives in intelligent active packages.

## Data Availability

Data are contained within the article.

## References

[1] Jung S., Cui Y., Barnes M., Satam C., Zhang S., Chowdhury R.A., Adumbumkulath A., Sahin O., Miller C., Sajadi S.M. (2020). Multifunctional Bio-Nanocomposite Coatings for Perishable Fruits. Adv. Mater..

[2] UNICEF (2022). World Food Programme: The State of Food Security and Nutrition in the World 2022.

[3] Jiang F., Zhou L., Zhou W., Zhong Z., Yu K., Xu J., Zou L., Liu W. (2022). Effect of modified atmosphere packaging combined with plant essential oils on preservation of fresh-cut lily bulbs. LWT.

[4] Qu P., Zhang M., Fan K., Guo Z. (2022). Microporous modified atmosphere packaging to extend shelf life of fresh foods: A review. Crit. Rev. Food Sci. Nutr..

[5] Zhao H., Jiao W., Cui K., Fan X., Shu C., Zhang W., Cao J., Jiang W. (2019). Near-freezing temperature storage enhances chilling tolerance in nectarine fruit through its regulation of soluble sugars and energy metabolism. Food Chem..

[6] Maringgal B., Hashim N., Tawakkal I.S.M.A., Mohamed M.T.M. (2020). Recent advance in edible coating and its effect on fresh/fresh-cut fruits quality. Trends Food Sci. Technol..

[7] Fu M.-R., Zhang X.-M., Jin T., Li B.-Q., Zhang Z.-Q., Tian S.-P. (2019). Inhibitory of grey mold on green pepper and winter jujube by chlorine dioxide (ClO_2_) fumigation and its mechanisms. LWT.

[8] Mei J., Ma X., Xie J. (2019). Review on Natural Preservatives for Extending Fish Shelf Life. Foods.

[9] Bakkali F., Averbeck S., Averbeck D., Idaomar M. (2008). Biological effects of essential oils—A review. Food Chem. Toxicol..

[10] Falleh H., Ben Jemaa M., Saada M., Ksouri R. (2020). Essential oils: A promising eco-friendly food preservative. Food Chem..

[11] Hyldgaard M., Mygind T., Meyer R.L. (2012). Essential oils in food preservation: Mode of action, synergies, and interactions with food matrix components. Front. Microbiol..

[12] Mutlu-Ingok A., Devecioglu D., Dikmetas D.N., Karbancioglu-Guler F., Capanoglu E. (2020). Antibacterial, Antifungal, Antimycotoxigenic and Antioxidant Activities of Essential Oils: An Updated Review. Molecules.

[13] Karoui R., Hassoun A. (2019). Efficiency of Rosemary and Basil Essential Oils on the Shelf-Life Extension of Atlantic Mackerel (*Scomber scombrus*) Fillets Stored at 2 °C. J. AOAC Int..

[14] Sugumar S., Ghosh V., Mukherjee A., Chandrasekaran N. (2016). Essential oil-based nanoemulsion formation by low-and high-energy methods and their application in food preservation against food spoilage microorganisms. Essential Oils in Food Preservation, Flavor and Safety.

[15] Ju J., Xie Y., Guo Y., Cheng Y., Qian H., Yao W. (2020). Application of starch microcapsules containing essential oil in food preservation. Crit. Rev. Food Sci. Nutr..

[16] Prakash B., Kujur A., Yadav A., Kumar A., Singh P.P., Dubey N.K. (2018). Nanoencapsulation: An efficient technology to boost the antimicrobial potential of plant essential oils in food system. Food Control.

[17] Pinto L., Tapia-Rodríguez M.R., Baruzzi F., Ayala-Zavala J.F. (2023). Plant Antimicrobials for Food Quality and Safety: Recent Views and Future Challenges. Foods.

[18] Mukurumbira A.R., Shellie R.A., Keast R., Palombo E.A., Jadhav S.R. (2022). Encapsulation of essential oils and their application in antimicrobial active packaging. Food Control.

[19] Zhang X., Ismail B.B., Cheng H., Jin T.Z., Qian M., Arabi S.A., Liu D., Guo M. (2021). Emerging chitosan-essential oil films and coatings for food preservation—A review of advances and applications. Carbohydr. Polym..

[20] Chen H.-Z., Zhang M., Bhandari B., Guo Z. (2018). Applicability of a colorimetric indicator label for monitoring freshness of fresh-cut green bell pepper. Postharvest Biol. Technol..

[21] Wang W., Zhao X., Xia Y., Xue Y., Cheng J., Yang F., Cui Y., Chen X., Wang R., Li X. (2023). Sugarcane-derived Bio-Amine-Responsive Colorimetric Films for real-time visual monitoring of the seafood freshness. Ind. Crops Prod..

[22] Kuswandi B., Jayus, Oktaviana R., Abdullah A., Heng L.Y. (2014). A Novel On-Package Sticker Sensor Based on Methyl Red for Real-Time Monitoring of Broiler Chicken Cut Freshness. Packag. Technol. Sci..

[23] Antony R., Arun T., Manickam S.T.D. (2019). A review on applications of chitosan-based Schiff bases. Int. J. Biol. Macromol..

[24] Heras-Mozos R., López-Carballo G., Hernández R., Gavara R., Muñoz P.H. (2023). pH modulates antibacterial activity of hydroxybenzaldehyde derivatives immobilized in chitosan films via reversible Schiff bases and its application to preserve freshly-squeezed juice. Food Chem..

[25] Barbosa H.F.G., Attjioui M., Leitao A., Moerschbacher B.M., Cavalheiro E.T.G. (2019). Characterization, solubility and biological activity of amphihilic biopolymeric Schiff bases synthesized using chitosans. Carbohydr. Polym..

[26] Higueras L., López-Carballo G., Gavara R., Hernández-Muñoz P. (2015). Reversible covalent immobilization of cinnamaldehyde on chitosan films via schiff base formation and their application in active food packaging. Food Bioprocess Technol..

[27] Lin J., Tang Z.-S., Brennan C.S., Chandrapala J., Gao W., Han Z., Zeng X.-A. (2023). Valorizing protein-polysaccharide conjugates from sugar beet pulp as an emulsifier. Int. J. Biol. Macromol..

[28] Lin J., Tang Z.-S., Brennan C.S., Zeng X.-A. (2022). Thermomechanically micronized sugar beet pulp: Dissociation mechanism, physicochemical characteristics, and emulsifying properties. Food Res. Int..

[29] Wu K., Zhang T., Chai X., Duan X., He D., Yu H., Liu X., Tao Z. (2023). Encapsulation Efficiency and Functional Stability of Cinnamon Essential Oil in Modified β-cyclodextrins: In Vitro and In Silico Evidence. Foods.

[30] Geng H., Yu X., Lu A., Cao H., Zhou B., Zhou L., Zhao Z. (2016). Extraction, Chemical Composition, and Antifungal Activity of Essential Oil of Bitter Almond. Int. J. Mol. Sci..

[31] Yeh C.H., Chou C.Y., Wu C.S., Chu L.P., Huang W.J., Chen H.C. (2022). Effects of Different Extraction Methods on Vanilla Aroma. Molecules.

[32] Si L., Chen Y., Han X., Zhan Z., Tian S., Cui Q., Wang Y. (2012). Chemical composition of essential oils of Litsea cubeba harvested from its distribution areas in China. Molecules.

[33] Huang X.-W., Feng Y.-C., Huang Y., Li H.-L. (2013). Chemical composition, antioxidant and the possible use as skin-care ingredient of clove oil (*Syzygium aromaticum* (L.) Merr. & Perry) and citronella oil (*Cymbopogon goeringii*) from China. J. Essent. Oil Res..

[34] Chen H., Zhao R., Hu J., Wei Z., McClements D.J., Liu S., Li B., Li Y. (2020). One-Step Dynamic Imine Chemistry for Preparation of Chitosan-Stabilized Emulsions Using a Natural Aldehyde: Acid Trigger Mechanism and Regulation and Gastric Delivery. J. Agric. Food Chem..

[35] Kotnik T., Žerjav G., Pintar A., Žagar E., Kovačič S. (2022). Azine-and imine-linked conjugated polyHIPEs through Schiff-base condensation reaction. Polym. Chem..

[36] Chen H., Hu X., Chen E., Wu S., McClements D.J., Liu S., Li B., Li Y. (2016). Preparation, characterization, and properties of chitosan films with cinnamaldehyde nanoemulsions. Food Hydrocoll..

[37] Lin J., Tang Z.-S., Chandrapala J., Brennan C.S., Han Z., Zeng X.-A. (2023). Elucidation of the cellulose nanocrystal-sugar beet pectin interactions for emulsification enhancement. Food Hydrocoll..

[38] Barbon S.M., Staroverov V.N., Gilroy J.B. (2015). Effect of extended π conjugation on the spectroscopic and electrochemical properties of boron difluoride formazanate complexes. J. Org. Chem..

[39] Lei X., Huang Y., Liang S., Zhao X., Liu L. (2020). Preparation of highly transparent, room-temperature self-healing and recyclable silicon elastomers based on dynamic imine bond and their ion responsive properties. Mater. Lett..

[40] Gleiter R., Haberhauer G. (2012). Aromaticity and Other Conjugation Effects.

[41] Chen H., McClements D.J., Chen E., Liu S., Li B., Li Y. (2017). In Situ Interfacial Conjugation of Chitosan with Cinnamaldehyde during Homogenization Improves the Formation and Stability of Chitosan-Stabilized Emulsions. Langmuir.

[42] Lin J., Yu S., Ai C., Zhang T., Guo X. (2020). Emulsion stability of sugar beet pectin increased by genipin crosslinking. Food Hydrocoll..

[43] Peng X., Liu P., Pang B., Yao Y., Wang J., Zhang K. (2019). Facile fabrication of pH-responsive nanoparticles from cellulose derivatives via Schiff base formation for controlled release. Carbohydr. Polym..

[44] Li J., Fu J., Tian X., Hua T., Poon T., Koo M., Chan W. (2022). Characteristics of chitosan fiber and their effects towards improvement of antibacterial activity. Carbohydr. Polym..

[45] Fontana R., Marconi P.C.R., Caputo A., Gavalyan V.B. (2022). Novel Chitosan-Based Schiff Base Compounds: Chemical Characterization and Antimicrobial Activity. Molecules.

[46] Shen S., Zhang T., Yuan Y., Lin S., Xu J., Ye H. (2015). Effects of cinnamaldehyde on *Escherichia coli* and *Staphylococcus aureus* membrane. Food Control.

[47] Guandalini Cunha B., Duque C., Sampaio Caiaffa K., Massunari L., Araguê Catanoze I., dos Santos D.M., de Oliveira S.H.P., Guiotti A.M. (2020). Cytotoxicity and antimicrobial effects of citronella oil (*Cymbopogon nardus*) and commercial mouthwashes on *S. aureus* and *C. albicans* biofilms in prosthetic materials. Arch. Oral. Biol..

[48] Porfirio E.M., Melo H.M., Pereira A.M.G., Cavalcante T.T.A., Gomes G.A., de Carvalho M.G., Costa R.A., Junior F. (2017). In Vitro Antibacterial and Antibiofilm Activity of Lippia alba Essential Oil, Citral and Carvone against *Staphylococcus aureus*. Sci. World J..

[49] Techavuthiporn C., Nakano K., Maezawa S. (2008). Prediction of ascorbic acid content in broccoli using a model equation of respiration. Postharvest Biol. Technol..

[50] Xu D., Zuo J., Fang Y., Yan Z., Shi J., Gao L., Wang Q., Jiang A. (2021). Effect of folic acid on the postharvest physiology of broccoli during storage. Food Chem..

[51] Jiang Y., Zhao D., Sun J., Luo X., Li H., Sun X., Zheng F. (2019). Analysis of antioxidant effect of two tripeptides isolated from fermented grains (Jiupei) and the antioxidative interaction with 4-methylguaiacol, 4-ethylguaiacol, and vanillin. Food Sci. Nutr..

